# The effect of aquatic exercise on bone mineral density in older adults. A systematic review and meta-analysis

**DOI:** 10.3389/fphys.2023.1135663

**Published:** 2023-03-13

**Authors:** Eileen Schinzel, Stephanie Kast, Matthias Kohl, Simon von Stengel, Franz Jakob, Katharina Kerschan-Schindl, Bernd Kladny, Uwe Lange, Stefan Peters, Friederike Thomasius, Jürgen Clausen, Michael Uder, Wolfgang Kemmler

**Affiliations:** ^1^ Institute of Radiology, FAU-Erlangen-Nürnberg and University Hospital Erlangen, Erlangen, Germany; ^2^ Research Group on Guideline “Exercise and Fracture Prevention”, Frankfurt, Germany; ^3^ Department of Medical and Life Sciences, University of Furtwangen, Schwenningen, Germany; ^4^ Bernhard-Heine-Centrum für Bewegungsforschung, University of Würzburg, Würzburg, Germany; ^5^ Austrian Society for Bone and Mineral Research, Vienna, Austria; ^6^ German Society for Orthopaedics and Trauma (DGOU), Berlin, Germany; ^7^ German Society for Physical and Rehabilitative Medicine, Leipzig, Germany; ^8^ German Association for Health-Related Fitness and Exercise Therapy (DVGS), Hürth, Germany; ^9^ Osteology Umbrella Association Germany, Austria, Switzerland; ^10^ Frankfurt Center of Bone Health, Frankfurt, Germany; ^11^ Deutsche Rheuma-Liga Bundesverband eV, Bonn, Germany; ^12^ Institute of Medical Physics, Friedrich-Alexander University of Erlangen-Nürnberg, Erlangen, Germany

**Keywords:** aquatic exercise, water-based exercise, bone mineral density, systematic review, meta-analysis

## Abstract

**Introduction:** Aquatic or water-based exercise is a very popular type of exercise in particular for people with physical limitations, joint problems and fear of falling. The present systematic review and meta-analysis aimed to provide evidence for the effect of aquatic exercise on Bone Mineral Density (BMD) in adults.

**Methods:** A systematic literature search of five electronic databases (PubMed/MEDLINE, Cochrane Library, Scopus, Web of Science and CINAHL) according to PRISMA (Preferred Reporting Items for Systematic Reviews and Meta-Analyses) was conducted until 2022/01/30, with an update to 2022/10/07. We included controlled trials with a duration of more than 6 months and at least two study groups, aquatic exercise (EG) versus non-training controls (CG) with no language restrictions. Outcome measures were standardized mean differences (SMD) with 95%-confidence intervals (95%-CI) for BMD changes at the lumbar spine (LS) and femoral neck (FN). We applied a random-effects meta-analysis and used the inverse heterogeneity (IVhet) model to analyze the data.

**Results:** Excluding an outlier study with an exceptionally high effect size for LS-BMD, we observed a statistically significant (*p* = .002) effect (EG vs. CG) of aquatic exercise for the LS-BMD (n = 10; SMD: 0.30; 95%-CI: 0.11–0.49). In parallel, the effect of aquatic exercise on FN-BMD was statistically significant (*p* = .034) compared to the CG (n = 10; SMD: 0.76, 95%-CI: 0.06–1.46). Of importance, heterogeneity between the trial results was negligible for LS (I^2^: 7%) but substantial for FN-BMD (I^2^: 87%). Evidence for risks of small study/publication bias was low for LS-BMD and considerable for FN-BMD.

**Discussion:** In summary, the present systematic review and meta-analysis provides further evidence for the favorable effect of exercise on bone health in adults. Due to its safety and attractiveness, we particularly recommend water-based exercise for people unable, afraid or unmotivated to conduct intense land-based exercise programs.

## 1 Introduction

Exercise is a highly effective tool for decreasing the risk of fragility fractures in older adults (Hoffmann et al., 2022). However the application of intense weight bearing (WBE) and dynamic resistance exercise (DRT) predominately recommended for increasing bone strength (DVO, 2009; Beck et al., 2016; Daly et al., 2019; Kemmler and Stengel, 2019) often conflicts with the physical situation and preferences of many older and/or vulnerable cohorts (Rodrigues et al., 2017). In addition, the risk of falling, frequently prevalent during intense WBE, discourages older people from starting and maintaining exercise (Rodrigues et al., 2017). Correspondingly, for people unable, afraid or unmotivated to exercise conventionally, aquatic/water-based interventions with their reduced stress on the joints, analgesic effect (Falagas et al., 2009) and lack of fall risk might be a suitable option. On the other hand, exercise categories without relevant weight-bearing character, e.g., swimming (Gomez-Bruton et al., 2013; Mohr et al., 2015; Gomez-Bruton et al., 2016; Su et al., 2020) or cycling (Nagle and Brooks, 2011; Olmedillas et al., 2012) failed to generate positive effects on Bone Mineral Density (BMD) at the lumbar spine (LS) or proximal femur (FN), i.e., the most important sites for osteoporotic fractures (Kanis et al., 2008). Of note however, the characteristics of aquatic exercise differ greatly from swimming. In this context, aquatic exercise should not be considered a rigid exercise categorization, but more as a vehicle for several types of exercise that are executed in chest high, predominately suitably heated water (Torres-Ronda and Del Alcazar, 2014). Apart from a few studies that focus on aerobic (aquatic) exercise (e.g., (Tsukahara et al., 1994; Pernambuco et al., 2013), the majority of studies applied resistance type exercise using water resistance to increase exercise intensity (Rotstein et al., 2008; Borba-Pinheiro et al., 2010; Borba-Pinheiro et al., 2012; Moreira et al., 2014; Wochna et al., 2019). Considering further that many studies (e.g., (Wochna et al., 2019) used devices (e.g., boards or cuffs) to increase water resistance during the movements, exercise intensity might be similar to intense conventional DRT. In their systematic review and meta-analysis, Simas et al. (2017) reported (statistically significant) higher effects of land-based versus water-based exercise on BMD in middle aged and older adults. However, more importantly, compared to sedentary controls, BMD effects of aquatic exercise were significantly higher at LS (mean difference 0.03 g/cm^2^; 95%-CI: 0.01–0.05 g/cm^2^) and FN (0.04 g/cm^2^; 95%-CI: 0.02–0.07 g/cm^2^). The authors (Simas et al., 2017) included six and five studies that focus on LS or femoral neck BMD respectively, however, several new trials on aquatic exercise were published after the search deadline of this work. Thus, the aim of this systematic review and meta-analysis was to provide an update on the effect of aquatic exercise on BMD at the lumbar spine and proximal femur region of interest (ROI). We hypothesize that aquatic exercise statistically significantly increases BMD at the LS and proximal femur (i.e., total hip or femoral neck ROI) compared with non-training controls.

## 2 Methods

This meta-analysis was reported according to the Preferred Reporting Items for systematic reviews and meta-Analyses (PRISMA) 2020 statement (Page et al., 2021). The present meta-analysis was registered on the International Prospective Register of Systematic Reviews (PROSPERO) under ID CRD42022298321.

### 2.1 Information sources

An overall search was performed on five electronic databases (PubMed/MEDLINE, Cochrane Library, Scopus, Web of Science and CINAHL) for all articles published from inception up to 2022/01/30 (with an update on 2022/10/07) with no language restrictions. Two articles with Spanish (Ramirez-Villada et al., 2016) or Japanese (Wu et al., 2000) full-text articles were translated by electronic resources (DeepL-translator).

### 2.2 Eligibility criteria

Inclusion criteria: 1) Adult participants of both genders. 2) Studies with participants on pharmaceutic osteoporosis therapy, when the number of subjects was comparable (difference <10%) between the exercise and control group. 3) Randomized and non-randomized controlled trials with at least one exercise group compared with a non-training control group. 4) A minimum of 6 months aquatic exercise intervention duration (shorter studies might not reach the full amount of mineralized bone and thus confound the BMD assessment). 5) Bone mineral density (BMD) of the lumbar spine (LS), femoral neck (FN) and/or total hip (TH) region at baseline and follow-up assessment as determined by 6) dual-energy x-ray absorptiometry (DXA), dual photon absorptiometry (DPA) or quantitative computed tomography (QCT). 7) Studies with pharmaceutic therapy on osteoporosis were included, however, only when exercise and control group were similarly provided.

Exclusion criteria: Studies that focus on 1) professional athletes or 2) animals. 3) Diseases or conditions that relevantly affect bone metabolism. 4) Double/multiple publications from one study (we included the publication with the most recent data on BMD) and preliminary data from subsequently published trials 5) Review articles, meta-analyses, case reports, editorials, conference abstracts, and letters.

### 2.3 Literature search

A standard protocol for this search was developed and a controlled vocabulary (MeSH term for MEDLINE, CINAHL^®^ Subject Headings for CINAHL) was applied. Keywords and their synonyms were used by applying the following queries (“water sports” OR “aquatics” OR “water aerobics” OR “warm water exercise” OR “aquatic weight-bearing exercises” OR “aquatic therapy” OR “hydro-gymnastics” OR “hydrogymnastics” OR “water exercise therapy” OR “water-based exercise” OR “exercise in water” OR “aqua* exercise” OR „aqua* gymnastics” OR “aqua* sports” OR “water exercise” OR “water based exercise” OR “water gymnastics” OR “hydro gymnastics” OR “swimming” OR “hydrotherapy” OR “exercise therapy“) AND (“bone strength” OR “BMC” OR “bone loss” OR “bone content” OR “bone*" OR “bone mass” OR “bone status” OR “bone structure” OR “bone turnover” OR “bone metabolism” OR “bone mineral content” OR “skeleton” OR “bone mineral density” OR “BMD” OR “bone density” OR “osteoporoses” OR “osteoporosis” OR “osteopenia")

Reference lists of eligible articles or reviews (Simas et al., 2017) dealing with the effect of aquatic exercise on BMD in adults were also screened. Articles were excluded when no full text was available or the reports were unpublished. We contacted authors by email when relevant data on eligibility, methods or results were unclear or missing.

### 2.4 Data extraction

ES and SK independently reviewed titles and abstracts for eligible articles, then the full-text articles were checked by ES and SK. Study data were extracted by ES, SK and WK with any disagreement being resolved by discussion. Data from included articles were checked using a extraction form that determined: a) publication details (e.g., first author’s name, publication year, country, study design); b) participant characteristics (gender, age, age at menopause, medication, diseases, medical conditions, bone status, lifestyle including Vitamin D and calcium intake and supplementation, physical activity, training status, body mass, body-height, BMI); c) study characteristics (length of the study, initial sample size, loss to follow-up); d) exercise characteristics including intervention length, type of exercise, exercise parameters (exercise frequency, intensity and volume), intensity progression, periodization, adherence, number of withdrawals, supervision of the session, adverse effects and f) supplementation with nutritional complements.

### 2.5 Outcome measures

Outcomes of interest were change in (areal) Bone Mineral Density at the lumbar spine (LS) and/or total hip (TH) region, or femoral neck (FN) assessed by DXA, DPA or QCT, between baseline and study end. Of importance, due to the aspect that most authors reported either TH or FN we had to conduct a joint analysis. In detail, TH BMD data were preferentially included in the analysis, however in cases where TH results were not reported, FN-BMD data of the studies were used. Nevertheless data were summarized under “FN”.

### 2.6 Quality assessment

To evaluate the methodologic quality of the trial, the articles were assessed independently by two reviewers (ES and SK) utilizing the PEDro (Physiotherapy Evidence Database scale risk of bias tool) (Sherrington et al., 2000) and TESTEX (Smart et al., 2015) score, specifically dedicated to physiotherapy (PEDro) and exercise (TESTEX) trials. In cases of inconsistency, a third reviewer decided (WK).

### 2.7 Data synthesis

Missing standard deviations (SD) were calculated using the method detailed in the recently published comprehensive meta-analysis by Shojaa et al. (Shojaa et al., 2020). If the studies presented a confidence interval (CI) or standard errors (SE), they were converted to standard deviation (SD) with standardized formulas (Higgins et al., 2011; Higgins et al., 2021). The subgroup analyses focused on differences in study length (i.e., <8 months versus ≥8 months).

### 2.8 Statistical analysis

We applied a random-effects meta-analysis using the metafor package (Viechtbauer, 2010) that is included in the statistical software R (R_Development_Core_Team, 2020). Effect size (ES) values were presented as standardized mean differences (SMDs) with a 95% confidence interval (95%-CI). We applied the heterogeneity (IVhet) model proposed by Doi et al. (2015). Heterogeneity between the studies was checked using *I* ^*2*^ statistics with *I* ^*2*^ categorization of 0%–40% being considered “low”, 30%–60% “moderate”, 50%–90% “substantial” and 75%–100% considerable heterogeneity (Higgins et al., 2011). Along with funnel plots, regression test and the rank correlation effect estimates and their standard errors using the *t*-test and Kendall’s τ statistic for potential publication bias, we also conducted a trim and fill analysis using the L0 estimator proposed by Duval et al. (Duval and Tweedie, 2000). Additionally, we used Doi plots and the Luis Furuya-Kanamori index (LFK index) (Furuya-Kanamori et al., 2017) to check for asymmetry. Sensitivity analyses were applied to determine whether the overall result of the analysis is robust to the use of the imputed correlation coefficient (minimum, mean or maximum). Furthermore, we applied a sensitivity analysis without an outlying study (Ramirez-Villada et al., 2016) due to doubt as to whether the authors reported standard deviation or standard error. *p*-value <0.05 was considered as the significance level for all tests. SMD values of 0.2, 0.5, and 0.8 were interpreted as small, medium, and large effects.

## 3 Results

**FIGURE 1:**
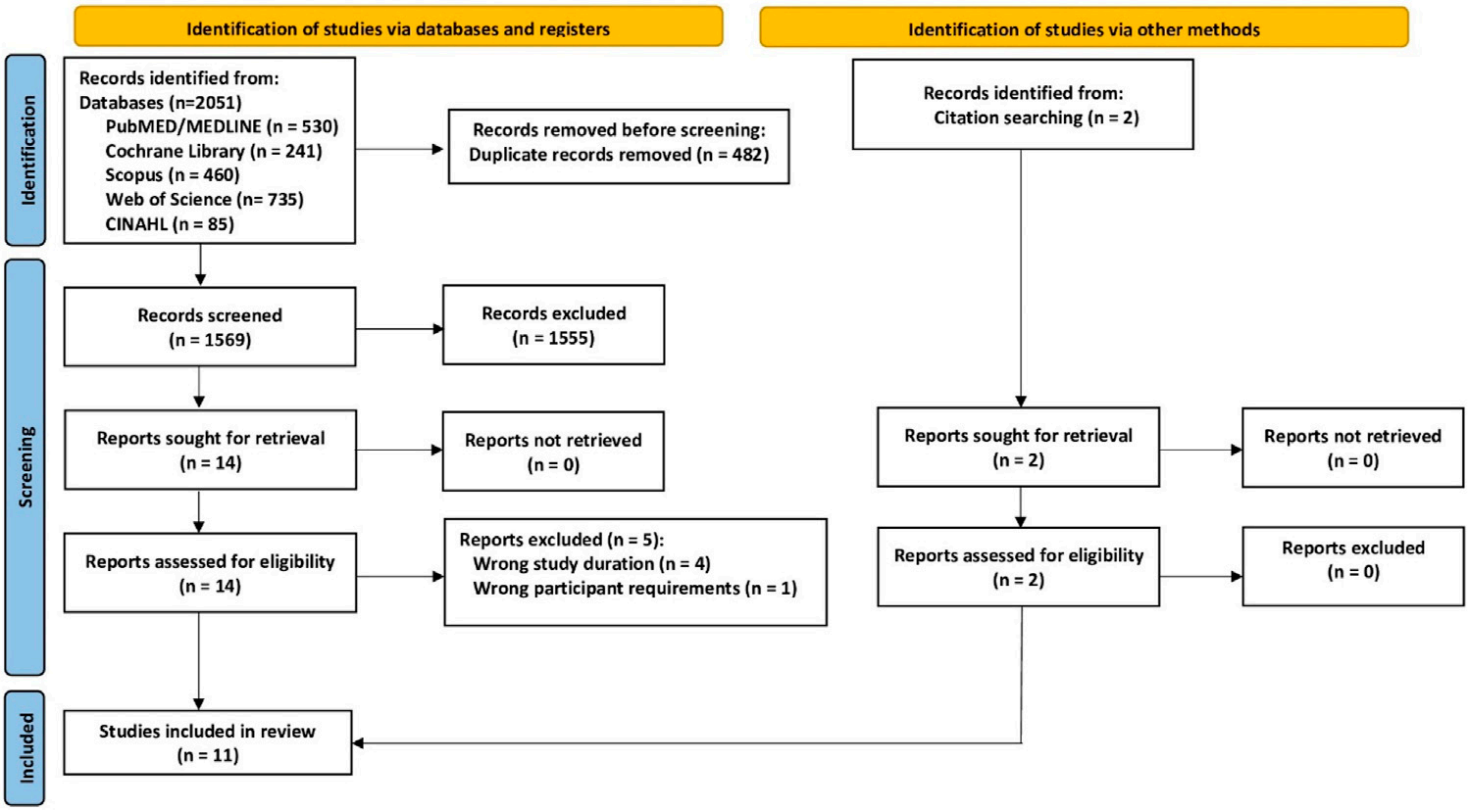
Flow chart of the present study according to PRISMA. Adapted from (Page et al., 2021).

Our search identified eleven eligible studies (Tsukahara et al., 1994; Wu et al., 2000; Littrell, 2004; Rotstein et al., 2008; Borba-Pinheiro et al., 2010; Borba-Pinheiro et al., 2012; Pernambuco et al., 2013; Moreira et al., 2014; Ramirez-Villada et al., 2016; Aboarrage Junior et al., 2018; Wochna et al., 2019) with eleven exercise and eleven non-training control groups each (Figure 1; Tables 1–3). The majority of studies (7 of 11) were non-randomized controlled trials (Tsukahara et al., 1994; Wu et al., 2000; Littrell, 2004; Rotstein et al., 2008; Borba-Pinheiro et al., 2010; Ramirez-Villada et al., 2016; Wochna et al., 2019). Sample size of the groups ranged from 7 (Borba-Pinheiro et al., 2010) to 64 participants/group (Moreira et al., 2014). The pooled number of included participants was 281 in the exercise and 274 in the control group. All studies applied dual energy x-ray absorptiometry (DXA)-technique to determine BMD at the LS and total hip or femoral neck ROI. Five studies were conducted between 1994 and 2019 in Brazil (Borba-Pinheiro et al., 2010; Borba-Pinheiro et al., 2012; Pernambuco et al., 2013; Moreira et al., 2014; Aboarrage Junior et al., 2018), two studies in Japan (Tsukahara et al., 1994; Wu et al., 2000) and one study each in Colombia (Ramirez-Villada et al., 2016), Israel (Rotstein et al., 2008), Poland (Wochna et al., 2019), and the US (Littrell, 2004).

**TABLE 1:** Study and participant characteristics of the included studies.

First author year	Study design	Sample size (n)	Age (years)	Bone status	Menopausal age (years)	BMI (kg/m^2^)
Aboarrage Junior et al. (2018)	RCT	EG: 15	57–75	No musculo-skeletal conditions	n.g	EG: 30 ± 5
		CG: 10				CG: 27 ± 7
Borba-Pinheiro et al. (2010)	NRCT	EG: 8	EG: 57 ± 7	Osteopenia	EG: 4 ± 1	EG: 26^a^
		CG: 7	CG: 54 ± 4	Osteoporosis	CG: 3 ± 1	CG: 26
Borba-Pinheiro et al. (2012)	RCT	EG: 43	EG: 55 ± 6	Osteopenia	EG: 4 ± 1	EG: 27^a^
		CG:41	CG:54 ± 5	Osteoporosis	CG: 4 ± 1	CG: 27^a^
Littrell (2004)	NRCT	EG: 27	EG: 67 ± 9	n.g	EG: 21 ± 10	EG: 29^a^
		CG: 32	CG: 67 ± 11		CG: 18 ± 10	CG:25^a^
Moreira et al. (2014)	RCT	EG: 64	EG: 59 ± 7	Normal, Osteopenia, Osteoporosis	At least 5 years	EG: 30^a^
		CG: 44	CG:59 ± 6			CG: 31^a^
Pernambuco et al. (2013)	RCT	EG: 36	EG: 67 ± 4^C^	Low BMD	EG: 45 ± 2^b^ ^,^ ^c^	EG: 29 ± 3
		CG: 31	CG: 67 ± 3		CG: 45 ± 2^b^ ^,^ ^c^	CG: 24 ± 3
Ramirez-Villada et al. (2016)	NRCT	EG: 17	EG: 60 ± 4^C^	No musculo-skeletal conditions	n.g	EG: 28 ± 3^C^
		CG: 18	CG: 63 ± 4^C^			CG: 26 ± 3^C^
Rotstein et al. (2008)	NRCT	EG: 25	EG: 55 ± 4	No musculo-skeletal conditions	EG: 5 ± 6	EG: 29^a^
		CG: 10	CG: 56 ± 4		CG: 6 ± 4	CG: 27^a^
Tsukahara et al. (1994)	NRCT	EG: 15	EG: 62 ± 4	No musculo-skeletal conditions	EG: 51 ± 3^b^	EG: 23 ± 1
		CG: 30	CG: 60 ± 2		CG: 51 ± 3^b^	CG: 23 ± 2
Wochna et al. (2019)	NRCT	EG: 9	EG: 58 ± 3	No musculo-skeletal conditions	EG: 10 ± 4	EG: 27 ± 3
		CG: 9	CG: 60 ± 3		CG: 12 ± 6	CG: 29 ± 4
Wu et al. (2000)	NRCT	EG: 22	EG: 60 ± 6	n.g	EG: 50 ± 3^b^	EG: 23 ± 2
		CG: 19	CG: 59 ± 5		CG: 51 ± 4^b^	CG: 22 ± 2

^a^ Calculated based on reported study results on body height and mass.

^b^ Age at menopause.

^c^ Data of participants that finished the study.

CG, control group; EG, exercise group; n.g, not given; NRCT, non-randomized controlled trial; RCT: randomized controlled trial.

### 3.1 Participant characteristics

Table 1 displays participant characteristics of the studies. All the trials included only postmenopausal women and most of the studies focus on participants without musculoskeletal conditions. However, four studies (Borba-Pinheiro et al., 2010; Borba-Pinheiro et al., 2012; Pernambuco et al., 2013; Moreira et al., 2014) included exclusively or predominately participants with low BMD, osteopenia or osteoporosis. Age of the participants ranged between 54 ± 5 years (Borba-Pinheiro et al., 2010; Borba-Pinheiro et al., 2012) and 67 ± 11 years (Littrell, 2004). Correspondingly, menopausal age varied from the early menopausal period (Rotstein et al., 2008; Borba-Pinheiro et al., 2010; Borba-Pinheiro et al., 2012) to the late postmenopause (Littrell, 2004). Although not specifically stated in the articles, participants of most studies were overweight to obese (Tab.1) (Littrell, 2004; Rotstein et al., 2008; Borba-Pinheiro et al., 2010; Borba-Pinheiro et al., 2012; Moreira et al., 2014; Aboarrage Junior et al., 2018; Wochna et al., 2019).

#### 3.1.1 Pharmaceutic therapy, Vitamin-D and calcium supplementation

Independently of the study group (i.e., EG or CG), Borba-Pinheiro et al. (2010); Borba-Pinheiro et al. (2012) and Pernambuco et al. (Pernambuco et al., 2013) supplied alendronate therapy (70 mg/week) for participants with osteoporosis and/or Vit-D supplementation (5600 IU/week) for participants with low bone mass (Pernambuco et al., 2013).

### 3.2 Intervention characteristics

#### 3.2.2 Exercise characteristics

The length of the intervention varied from 6 (Moreira et al., 2014; Ramirez-Villada et al., 2016; Aboarrage Junior et al., 2018; Wochna et al., 2019) to 24 (Wu et al., 2000) months. Three studies did not report the pre-study exercise status of their participants (Tsukahara et al., 1994; Wu et al., 2000; Borba-Pinheiro et al., 2012). Only one study considered its cohort as being sedentary (Moreira et al., 2014) while Ramirez-Villada et al. (2016) included participants with exercise habits that might have affected the result of the later exercise intervention (Table 2). Although all studies applied “water-based exercise,” the type of exercise actually prescribed varied considerably. One study applied (not only but largely) swimming exercise (Wu et al., 2000), while most of the other studies applied a mix of aerobic, jumping and resistance exercise using water resistance to increase exercise intensity (Table 2). Eight trials scheduled jumps or other explosive movements (Littrell, 2004; Borba-Pinheiro et al., 2010; Borba-Pinheiro et al., 2012; Pernambuco et al., 2013; Moreira et al., 2014; Ramirez-Villada et al., 2016; Aboarrage Junior et al., 2018; Wochna et al., 2019). Specification of training frequency/week and volume/session varied from ≥1 session of 60 min (Tsukahara et al., 1994) to 3 sessions of 90 min (Ramirez-Villada et al., 2016) per week (Tab. 2). Unfortunately, four studies (Wu et al., 2000; Littrell, 2004; Ramirez-Villada et al., 2016; Wochna et al., 2019) did not state exercise intensity specification. Since intensity specification is based predominately on RPE prescription, it is difficult to categorize studies with intensity progression during the intervention (Table 2). Nevertheless, with respect to studies of 12 months and longer (Tsukahara et al., 1994; Wu et al., 2000; Littrell, 2004; Borba-Pinheiro et al., 2010; Borba-Pinheiro et al., 2012), i.e., studies with increased relevance of intensity progression, only two studies (Borba-Pinheiro et al., 2010; Borba-Pinheiro et al., 2012) reported a structured intensity progression. Further, only three trials (Littrell, 2004; Moreira et al., 2014; Ramirez-Villada et al., 2016) reported attendance rates, thus the net training frequency of most studies is not known. Finally, loss to follow-up ranges from 0% for the 24-month study of Wu et al. (2000) to 25% for the 12-month study of Littrell, (2004). However, loss of follow-up was not consistently reported by all the studies (Table 2). Nevertheless, based on attendance rates and drop-out/loss to follow-up (Table 2), aquatic exercise can be considered as an attractive type of exercise (Shojaa et al., 2020; Kistler-Fischbacher et al., 2021). Finally, four studies (Littrell, 2004; Moreira et al., 2014; Ramirez-Villada et al., 2016; Aboarrage Junior et al., 2018) (Table 3) listed adverse effects. In summary, no injuries, serious medical event or other unintended side effects induced by the exercise protocol were reported.

**TABLE 2:** Exercise characteristics of the included studies.

First autor year	Intervention length (mo.)	Training status	Exercise/strain composition exercise frequency × duration of the session (in min/week), type of exercise, sets/repetitions or duration of the exercise, exercise intensity, movement velocity	Atten-dence rate (%)	Loss to follow up (%)
Aboarrage Junior et al. (2018)	6	Specifically untrained for 3 months	Water-based jump exercise (water depth n.g.): 3 × 30 min/w: 5 min warm-up/cool down with stretching and free movements, jump-based exercise (single leg, ankle hops, tuck jumps, jumps with hip abduction and adduction) with 20 sets of 30 s with high intensity (“all out”) and 30 s of passive recovery between the bouts; GRF n.g	n.g	0
Borba-Pinheiro et al. (2010)	12	Specifically untrained for 12 months	Water-based functional gymnastic and jumping (water depth 145 cm): 3 × 60 min/w.: bimonthly periodized resistance exercises, 8–12 exercises (balance body displacements, shoulder adduction/abduction, jumps with knee extension, knee flexion, elbow flexion/-extension, squats), 3 series of 6–20 reps at RPE 14–16 Borg CR20; all participants under Alendronate-therapy	n.g	n.g
Borba-Pinheiro et al. (2012)	12	n.g	Water-based functional gymnastic and jumping (145 cm): 3 × 60 min/w.: bimonthly periodized resistance exercises, 8–12 exercises (balance body displacements, shoulder adduction/abduction, jumps with knee extension, knee flexion, elbow flexion/-extension, squats), 3 series of 6–20 reps at RPE 14–16 Borg CR20; all participants under Alendronate-therapy	n.g	n.g
Littrell (2004)	12	Specifically untrained	Shallow water exercise (110–137 cm): 3 × 45 min/w.; warm up/cool down (walking/jogging, stretching), aerobics (jumping jacks, tuck jumps, jogging, rocks, leg curls, cross-country skis), one/two leg jumps, heel drops, muscle fitness/stability (upper body and trunk exercises), fall recovery/balance exercise; exercise intensity n.g	93	25
Moreira et al. (2014)	6	Sedentary	Aquatic exercise (110–130 cm): 3 × 50–60 min/w. with progressive exercise intensity: strength and power training: 2–5 sets 30–10 s with maximum movement speed at RPE 6–9 Borg CR10 with 60–100 s breaks; 16–7 min continuous (?) cardiorespiratory exercise at RPE 6–9 Borg CR10; 10 min of warm up/cool down	93	8
Pernambuco et al. (2013)	8	No regular exercise in last 6 months	Aquatic aerobic (140 cm): 2 × 50 min/w. warm up with stretching and leg and of arms movements; 5 sequences of 7 min: Stationary running, ski movements with trunk rotations, elbow and leg flexion/rotation, with and without barbells, jumps with hip hyperextension, plantar flexion, isometric contraction of the gluteus/quadriceps; exercise intensity was not reported	n.g	14
Ramirez-Villada et al. (2016)	6	2–3 exercise sessions/w	Aquatic exercise with explosive movements (water depth n.g.): 3 × 90 min/w. vertical and horizontal jumping, short sprints, 3 exercises with 2–3 sets of 8 reps each, 25–30 min of aerobic dance, exercise intensity n.g	77	12
Rotstein et al. (2008)	7	n.g	Aquatic exercise (chest level): 3 × 60 min; 10 min warm up, 20 min aerobic exercise 12–16 Borg CR20 (Borg and Kaijser, 2006), 20 min strengthening and “bone loading” using devices to increase water resistance and elastic bands, four movement patterns: compression, twisting, stretching/extension, bending/flexion; intensity n.g. 10 min of static and dynamic stretching	n.g	20
Tsukahara et al. (1994)	12	n.g	Aquatic aerobic exercise (water depth n.g.): ≥1 × 45 min/week; 10 min warm-up, 20 min of aerobic exercise and deep breathing (?), 10 min of swimming at ≈120 beats/min	n.a	n.g
Wochna et al. (2019)	6	No systematic physical activity	Aquatic fitness (deep water: neck line): 2 × 45 min/week with equipment that increased the water resistance; predominately movements to music in a vertical position; exercise intensity n.g	n.g	n.g
Wu et al. (2000)	24	n.g	Aquatic exercise, swimming (effectively) 1.5 × 60 min/week: 1,000 m swimming (5–6 × 150–200 m breaststroke, backstroke, crawl), water gymnastic, stretching, water walking; details n.g	n.g	0

CG, control group; EG, exercise group; GRF, ground reaction forces; n.g, not given; reps: repetition; RPE: rate of perceived exertion, w, week.

**TABLE 3:** Methodologic quality of the included studies.

	PEDro-criteria												Additional TESTEX criteria^a^					
Author, year	Eligibility criteria	Random allocation	Allocation concealment	Inter group homogeneity	Blinding subjects	Blinding personnel	Blinding assessors	participation≥ 85% allocation	Intention to treat analysis^b^	Between group comparison	Measure of variability	Total score PEDro	Adverse effects reported	Attendance reported	Activity monitoring in control groups	Relative exercise intensity constant	Exercise volume and energy expended	Total score TESTEX
Aboarrage Junior et al. (2018)	Y	+	—	+	—	—	+	+	+	+	+	7	+	—	—	—	—	10
Borba-Pinheiro et al. (2010)	Y	—	—	—	—	—	—	+	—	+	—	2	—	—	—	+	+	7^c^
Borba-Pinheiro et al. (2012)	Y	+	—	—	—	—	—	—	—	+	+	3	—	—	—	+	+	7
Littrell (2004)	Y	—	—	—	—	—	—	—	—	+	+	2	+	+	+	—	—	7
Moreira et al. (2014)	Y	+	—	+	—	—	—	+	—	+	+	5	+	+	+	+	+	12
Pernambuco et al. (2013)	Y	+	+	+	—	—	+	—	—	+	+	6	—	—	—	—	—	8
Ramirez-Villada et al. (2016)	Y	—	—	+	—	—	+	—	—	+	+	4	+	+	—	—	+	9
Rotstein et al. (2008)	Y	—	—	+	—	—	—	+	—	+	+	4	—	—	—	+	+	8
Tsukahara et al. (1994)	Y	—	—	+	—	—	—	—	—	—	+	2	—	—	—	—	—	3^c^
Wochna et al. (2019)	Y	—	—	+	—	—	—	—	—	+	+	3	—	—	—	—	—	5
Wu et al. (2000)	Y	—	—	+	—	—	—	+	+	+	+	5	—	—	+	—	—	8

^a^ TESTEX awards one point for listing the eligibility criteria and, also in contrast to PEDro, a further point for the between group comparison of at least one secondary outcome.

^b^ …. or all subjects received treatment or control as allocated (…or were retrospectively analyzed).

^c^ TESTEX awards one point if all outcomes are reported with point estimates, in contrast to PEDro which only awards one point if both point estimates and measures of variability.

### 3.3 Methodological quality

Table 3 shows the methodological quality of the included studies according to the PEDro (Sherrington et al., 2000) and TESTEX (Smart et al., 2015) score. Following PEDro and applying the classification of Ribeiro de Avila et al. (Ribeiro de Avila et al., 2018), the methodologic quality of seven studies can be classified as low (PEDro: <5) and four studies as moderate (PEDro: 5–7). In particular, aspects related to allocation concealment or blinding were not satisfied or not reported. Another important aspect that has been neglected by the vast majority of studies are adverse effects, i.e., exercise induced injuries, serious medical event or other unintended negative side effects.

### 3.4 Meta-analysis results

#### 3.4.1 BMD changes at the lumbar spine region of interest

**FIGURE 2:**
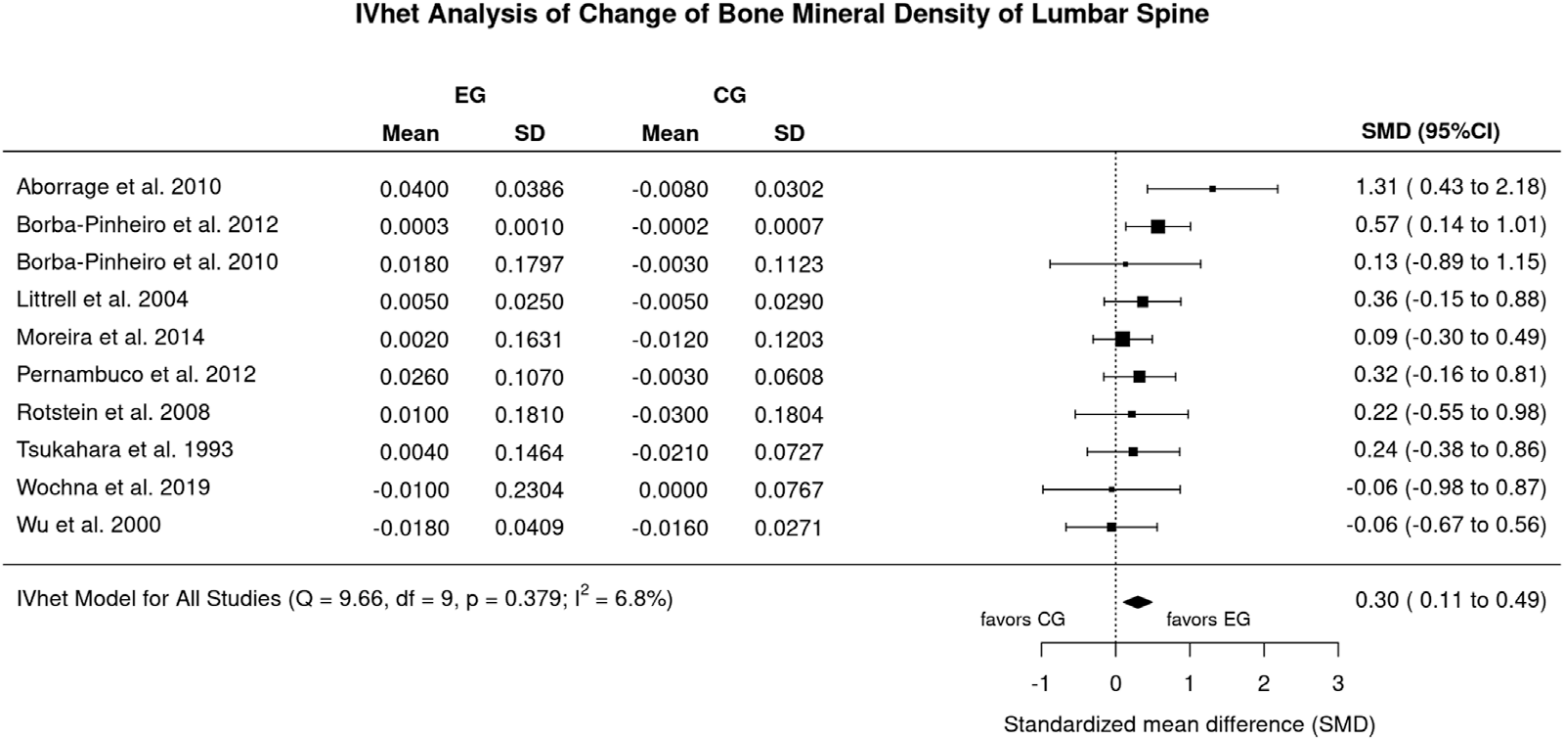
Forest plot of meta-analysis results of all included trials at the lumbar spine. Data shown as pooled standardized mean difference (SMD) with 95%-CI for changes in the, EG versus the CG.

Figure 2 displays the results of aquatic exercise versus CG on LS-BMD. Excluded an outlier with a exceptionally high effect size (SMD: 10.06; 96%-CI: 7.27–12.94) (Ramirez-Villada et al., 2016), we observed statistically significant effects (*p* = .002) of aquatic exercise on LS-BMD (SMD: 0.30; 95%-CI: 0.11–0.49) (Figure 2). Heterogeneity between the trials was negligible (*I* ^*2*^: 7%, Figure 2) Sensitivity analysis with respect to imputation of the mean correlation (see Figure 2), minimum or maximum correlation revealed roughly comparable effects.

**FIGURE 3:**
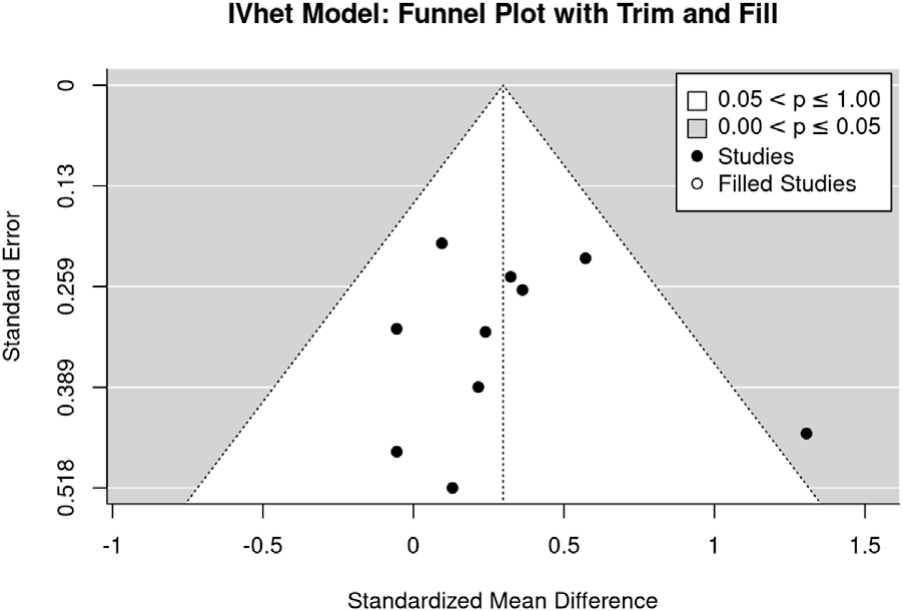
Funnel plot of the studies that address lumbar spine BMD.

The funnel plot with trim and fill analysis suggests no evidence for a publication/small study bias for LS-BMD (Figure 3). The LFK Index (0.39) confirmed the negligible asymmetry, in parallel the regression (*p* = .833) and rank correlation test (*p* = .727) do not indicate statistically significant asymmetry.

#### 3.4.2 BMD changes at the proximal femur region of interest

**FIGURE 4:**
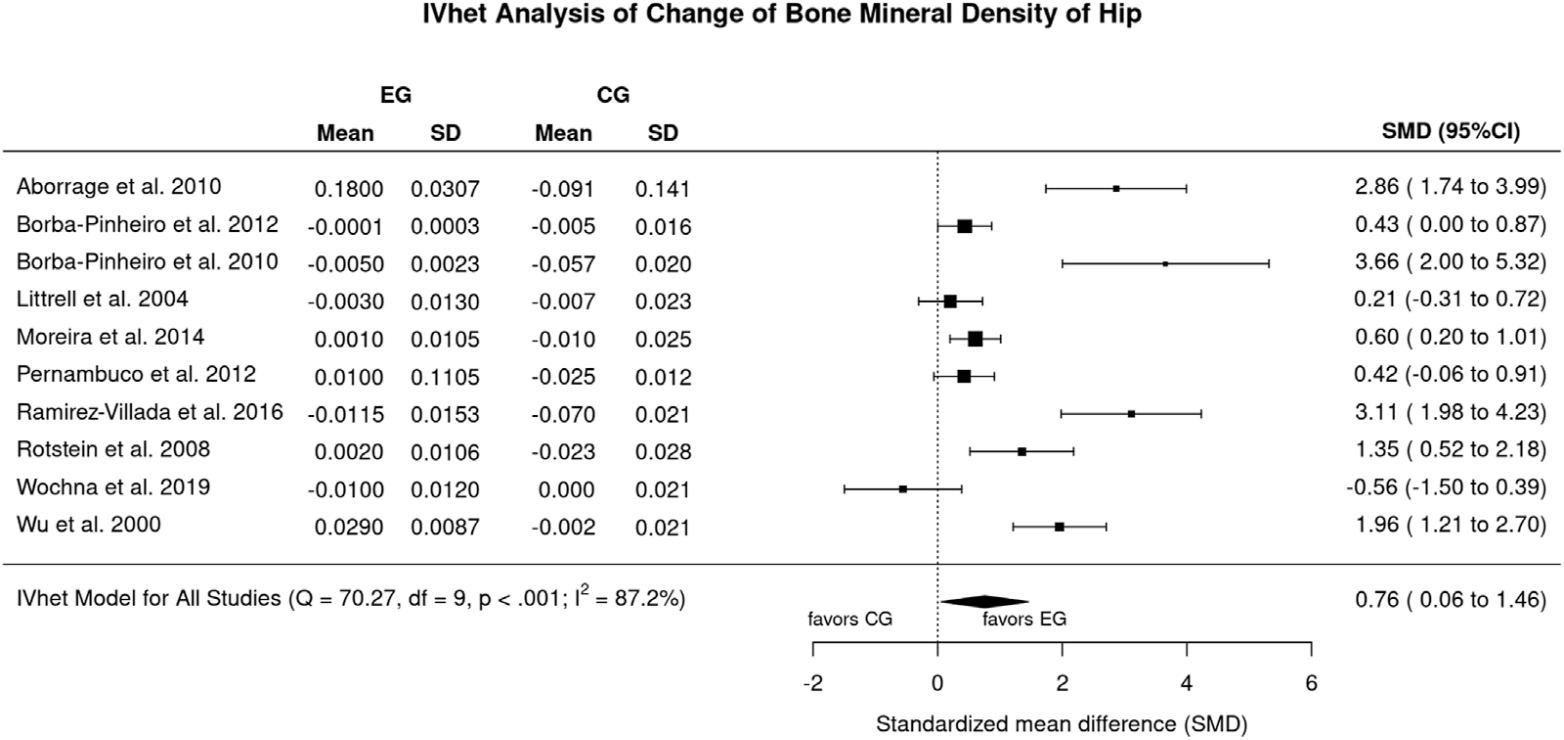
Forest plot of meta-analysis results for the proximal femur region of interest. Data shown as pooled standardized mean difference (SMD) with 95%-CI for changes in the EG versus the CG.

**FIGURE 5:**
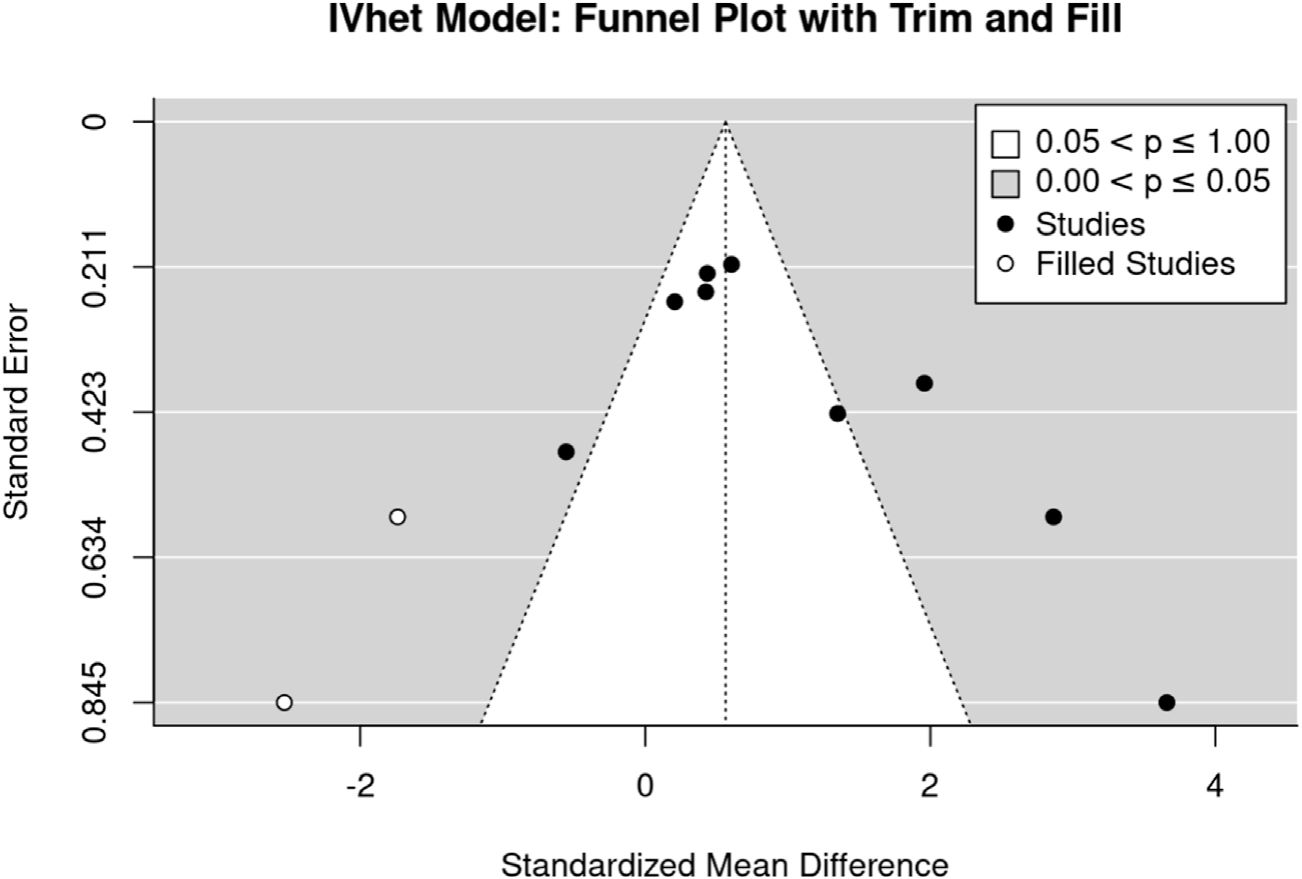
Funnel plot of the studies that address lumbar spine BMD.

Figure 5 displays the results on FN-BMD. In brief, the effect of aquatic exercise on FN-BMD was statistically significantly (*p* = .034) higher compared to the non-training CG (SMD: 0.76, 95%-CI: 0.06–1.46). Heterogeneity between the trial results was considerable (*I* ^*2*^: 87%; Figure 4). Sensitivity analysis with respect to imputation of the mean correlation (see Figure 4), minimum or maximum correlation revealed roughly comparable effects.

The funnel plot with trim and fill analysis suggests considerable evidence for a publication/small study bias for FN-BMD (Figure 5). Two missing studies on the lower left-hand side (i.e., small studies with negative outcome) were imputed. The LFK Index (3.73: major asymmetry) and the regression test (*p* = .020), but not the rank correlation test (*p* = .216), confirmed the statistically significant asymmetry of the plot.

#### 3.4.3 Subgroup-analysis on study length

Briefly, we did not determine any statistically significant differences between shorter and longer (5 comparisons each) study duration for the LS (*p* = .809) or FN BMD (*p* = .576). In detail, however, results on BMD were statistically significant only for studies ≥8 months.

## 4 Discussion

In summary, we verified our hypotheses through observing statistically significant effects of aquatic exercise for lumbar spine and proximal femur BMD. This finding is of particular importance since aquatic exercise is very popular in eastern and central Europe. In Germany alone roughly 15,000 aquatic exercise groups are run under the legal requirements of § 64, German Social Security Code IX (SGB_IX, 2019). Although the evidence for positive effects of aquatic exercise on BMD had been moderate at best, the majority of these mandatorily supervised aquatic exercise groups focus on participants with osteopenia and osteoporosis. Correspondingly, the result of the present systematic review and meta-analysis on aquatic exercise and bone health is of particular interest for the non-pharmacologic treatment of osteoporosis. We are not the first to report the favorable effect of water-based exercise on bone health. In their systematic review and meta-analysis of seven comparisons (aquatic exercise vs. sedentary control), Simas et al. (2017) observed statistically significant effects of water-based exercise on LS and FN-BMD. However, the authors also reported a statistically significant superiority of land-based versus water-based exercise programs (4 comparisons) on LS- and (less pronounced) FN-BMD. Less surprisingly, due to the buoyancy effect, peak vertical ground reaction forces of typical water exercises (e.g., stationary running, Nordic Skiing) were roughly half as high in the aquatic compared to the dry environment (Alberton et al., 2013). Undoubtedly, the main effect of aquatic exercise on bone is triggered by joint reaction forces, while the effect of ground reaction forces during (breast-high) water-based exercise is less relevant or even negligible (Alberton et al., 2013). On the other hand, the minor impact on lower back, hip and lower limb joints, analgesic effects (Hinman et al., 2007; Falagas et al., 2009), psychological comfort (Wochna et al., 2019) and negligible fall risk/fall consequences make aquatic exercise suitable for many people. This includes cohorts with osteoarticular limitations (Mattos de et al., 2016) or overweight (Torres-Ronda and Del Alcazar, 2014) but also physically limited older people.

While focusing exclusively on postmenopausal women and aquatic exercise, the included studies might be considered homogeneous at first. Nevertheless, considerable differences in participant (Table 1) and particularly in exercise characteristics (Table 2) might have confounded our results. Menopausal age (early vs. late postmenopausal) and bone status (normal BMD vs. osteopenia/osteoporosis), which vary widely between the studies (Table 1), might be candidates that modulate exercise effects on bone (Kemmler and Riedel, 1998; Kemmler, 1999). Having said that, two recent meta-analyses on exercise and BMD (Shojaa et al., 2020; Mohebbi et al., 2023) did not report statistically significant differences between the categories. Reviewing the exercise characteristics of the included studies was a daunting task because many studies (Table 2) did not completely or comprehensibly report their exercise protocols. We opt to focus our subgroup analysis on study length, as this was consistently reported by all trials and might be an important modulator of the exercise effects on BMD. Indeed, considering bone remodeling as the primary mode of bone renewal in adults (Eriksen, 2010; Erben, 2015), taking initial familiarization and conditioning phases and regular changes in exercise intensity into account, shorter studies might not reach the full amount of mineralized bone and thus confound the BMD assessment. Categorizing study length into <8 versus ≥8 months did not result in statistically significant differences between the subgroups, however. We attribute this result in part to the very complex interaction between types of exercise, exercise parameters and training principle that aggravates or even prevents a reliable subgroup-analysis of single exercise characteristics in comprehensive meta-analyses (Kemmler, 2013; Gentil et al., 2017).

Undoubtedly, our systematic review and meta-analysis feature some limitations and study particularities that should be considered to properly interpret our results. 1) First of all, we have to admit that we were not always convinced whether the studies were interventional (or prospective). This refers to the study of Tsukahara et al. (Tsukahara et al., 1994) and Wu et al. (Wu et al., 2000). After internal discussion, we finally included the studies due to the aspect that according to the authors, initial (BMD) assessment and start of the training program were closely related in time. 2) Related to this issue, unfortunately none of the authors who were contacted (*n* = 5) by email responded to resolve important methodological issues. This includes the simple issue as to whether variations for BMD changes were given as standard error (SE) or standard deviation (SD). 3) The exclusion of the trial of Ramirez-Villada et al. (2016) is primarily related to the very extreme SMD of 10.06 (7.27–12.84) (LS-BMD, Figure 2), which does not seem realistic to us. It might be caused by the possible confusion of standard deviation and standard error for LS-BMD by the authors. Including the study of Ramirez-Villada et al. (Ramirez-Villada et al., 2016) considerably increase heterogeneity (I^2^ = 82%) and lead to non-statistically significant results for LS-BMD (SMD: 0.34; 95%-CI: 0.18–0.96) 4) The methodological quality of the studies ranged between 2 and 7 of a maximum of 10 score-points (median 3.5) for PEDro (Sherrington et al., 2000) and 3 to 12 of a maximum of 15 score-points (median 7.5) for TESTEX (Smart et al., 2015), which on average is low. Even when considering that blinding of participants and caregivers (i.e., trainers) is hardly realizable in exercise studies, exercise trials in general should be more aware of reporting standards (e.g., CONSORT; (Moher et al., 2010) and methodological quality scores (PEDro; TESTEX) so as to ensure the adequacy and completeness of study information that is essential in a publication on exercise. 5) In this context, unfortunately some studies (Table 2) did not report drop-out or exercise attendance, two aspects that reflect the feasibility and attractiveness of the trainings protocol. 6) We included three studies (Borba-Pinheiro et al., 2010; Borba-Pinheiro et al., 2012; Pernambuco et al., 2013) that involved participants with Alendronate therapy. However, due to the aspect that, EG and CG were similarly supplemented and no additive or synergistic effects on exercise and Bisphosphonate therapy have been reported (Klotz et al., 2022), we do not expect a relevant confounding effect. 7) Due to the remodeling issue (Eriksen, 2010; Erben, 2015) discussed above, we include only studies with a minimum of 6 months intervention duration. 8) Due to the aspect that we observed relevant heterogeneity among the studies in a number of meta-analyses on training studies (Hamilton et al., 2021; Mohebbi et al., 2023), we performed a random-effects meta-analysis and specifically chose the applied the inverse heterogeneity model (IVhet) (Doi et al., 2015). This model is less prone to underestimating the statistical error and thus leads to confidence intervals that meet the specified coverage probability better. (Furuya-Kanamori et al., 2017). 9) All the trials included focus on postmenopausal women, thus the generalization of our results might be limited on this cohort.

Summing up our results on aquatic exercise and BMD, we provided further evidence for the positive effect of this training option specifically suitable for physically limited older cohorts with low physical fitness and at risk for falls. Nevertheless, some other important research questions on aquatic exercise should be answered in the near future. This relates in particular to the validation of its positive effects in other cohorts with increased fracture risk. Further, aquatic exercise studies with multiple exercise groups should address the effects of different exercise characteristics (e.g., low vs. high exercise intensity, frequency and duration) on BMD in order to provide validated recommendations on aquatic exercise programs.

## Data Availability

The original contributions presented in the study are included in the article/supplementary material, further inquiries can be directed to the corresponding author.
